# Contribution of Toxin–Antitoxin Systems to Adherent-Invasive *E. coli* Pathogenesis

**DOI:** 10.3390/microorganisms12061158

**Published:** 2024-06-06

**Authors:** Paula Bustamante, María Núria Ramos-Corominas, Margarita Martinez-Medina

**Affiliations:** 1 Molecular and Cellular Microbiology Laboratory, Instituto de Ciencias Biomédicas, Facultad de Ciencias de la Salud, Universidad Autónoma de Chile, Santiago 8910060, Chile; 2 Microbiology of Intestinal Diseases, Biology Department, Universitat de Girona, 17003 Girona, Spain; marianuria.ramos@udg.edu (M.N.R.-C.); marga.martinez@udg.edu (M.M.-M.)

**Keywords:** toxin–antitoxin systems, AIEC, Crohn’s disease, stress response, persistence, pathogenicity

## Abstract

Pathobionts have been implicated in various chronic diseases, including Crohn’s disease (CD), a multifactorial chronic inflammatory condition that primarily affects the gastrointestinal tract, causing inflammation and damage to the digestive system. While the exact cause of CD remains unclear, adherent-invasive *Escherichia coli* (AIEC) strains have emerged as key contributors to its pathogenesis. AIEC are characterized by their ability to adhere to and invade intestinal epithelial cells and survive and replicate inside macrophages. However, the mechanisms underlying the virulence and persistence of AIEC within their host remain the subject of intensive research. Toxin–antitoxin systems (TAs) play a potential role in AIEC pathogenesis and may be therapeutic targets. These systems generally consist of two components: a toxin harmful to the cell and an antitoxin that neutralizes the toxin’s effects. They contribute to bacterial survival in adverse conditions and regulate bacterial growth and behavior, affecting various cellular processes in bacterial pathogens. This review focuses on the current information available to determine the roles of TAs in the pathogenicity of AIEC. Their contribution to the AIEC stress response, biofilm formation, phage inhibition, the maintenance of mobile genetic elements, and host lifestyles is discussed.

## 1. Introduction

The discovery of novel targets for antimicrobial agents is essential to fight against bacterial pathogens that cause diverse pathologies, including chronic diseases. Toxin–antitoxin systems (TAs) are present in nearly all bacterial and archaeal strains, and they have emerged as potential virulence factors, as they not only affect pathogenicity but are also related to biofilm formation and persistence. In addition, they play a role in the stabilization of mobile genetic elements (MGEs) and stress response [1,2]. As a result, components of TAs have been recognized as promising therapeutic targets [3]. They were initially found to promote plasmid maintenance by selectively eliminating daughter cells that do not inherit a plasmid copy during cell division (i.e., PSK systems) [4], and their plasmid maintenance function has been well established. Subsequently, TA modules were found to be highly abundant in the chromosomes of almost all free-living bacteria, and despite their abundance and prevalence, their biological roles have remained poorly defined and even controversial [5].

Canonical TA modules consist of two genes in an operon: a stable toxin whose overexpression affects bacterial growth and a usually unstable antitoxin that neutralizes the toxin’s action. Based on the antitoxin mechanism, six major types of TAs are recognized. While almost all known TA toxins are proteins, antitoxins can be small RNAs that repress toxin protein expression by interacting with the toxin mRNA (type I) or sequester the toxin by direct binding (type III). Antitoxins can also be proteins that directly bind and inhibit the toxin (type II), function as a toxin antagonist (type IV), cleave the toxin mRNA (type V), or promote the degradation of the toxin serving as a ClpXP protease adaptor (type VI). However, there are examples of newly discovered TAs classified as type VII and VIII, in which the antitoxin chemically modifies the toxin post-translationally to neutralize it [6] or the toxin is a small RNA whose activity is masked by the antitoxin anti-sense binding [7], respectively. On the other hand, most toxins are enzymes that interfere with translation, although they can affect a wide variety of cellular processes such as DNA replication, cell division, and membrane stability [2].

Stresses to which TAs respond when they are upregulated and/or the toxin is activated are also stresses that are encountered by pathogens in different niches inside their hosts, such as nutrient starvation, bile salts, acidic pH, and oxidative stress [8,9,10]. Hence, TAs could modulate bacterial physiology and, consequently, play a crucial role in bacterial virulence and pathogenesis.

## 2. Crohn’s Disease and Adherent-Invasive *Escherichia coli*

Crohn’s disease (CD) is a subtype of inflammatory bowel disease (IBD) characterized by a severe and recurrent chronic immune-mediated granulomatous inflammation, which can affect any region of the gastrointestinal tract and cause diarrhea, intestinal bleeding, abdominal pain, anemia, and weight loss [11]. As a global disease, CD (and IBD in general) has increased its incidence worldwide in the 21st century, particularly in the Western hemisphere [12].

The etiology of CD has not been elucidated, although it is known to be a multifactorial process driven by an aberrant immune response to gut bacteria in a genetically susceptible host [13]. Microbial factors have proven to be indispensable for the onset of this disease. IBD patients share microbial patterns such as reduced microbial diversity, a decreased relative abundance of Firmicutes, and increased Proteobacteria [14]. Among the Proteobacteria, adherent-invasive *E. coli* (AIEC) are frequently isolated from CD patients [15,16], suggesting their role in disease development. Indeed, evidence was recently found of AIEC’s causal role in intestinal inflammation [17].

AIEC are a clonally diverse *E. coli* pathotype that genetically clusters with extraintestinal pathogenic *E. coli* (ExPEC) [18], but they can penetrate the mucin layer, adhere to and invade intestinal epithelial cells (IECs), translocate to the intestinal epithelium, and colonize macrophages [19,20,21,22]. While they lack the known virulence factors and invasive determinants of other *E. coli* pathotypes, their pathogenic mechanisms are not fully understood, and most of their virulence genes are not AIEC-specific [23,24]. The prototypes for AIEC are the *E. coli* LF82 [25] and NRG857c [18] strains, which are included in most studies analyzing *E. coli* strains associated with CD.

AIEC rely on metabolic adaptations to outcompete the native microbiota and successfully colonize the intestinal mucosa [23,24] and can modulate the immune responses to persist in the gut [26]. The pathogenic potential of AIEC manifests under certain host conditions, e.g., AIEC upregulates L-serine metabolism pathways in the inflamed gut to adapt to the inflammatory microenvironment [27]. Moreover, exposure to bile salts upregulates the expression of metabolic genes such as *eut* and *pdu*, allowing AIEC to metabolize 1,2-propanediol (*pdu* operon) and ethanolamine (*eut* operon) [23,28]. Consequently, the *pdu* and *eut* operons, which are both enriched in AIEC genomes [24], represent a metabolic adaptation that may foster AIEC blooms in the gut. In addition, the ability of AIEC to secrete mucinolytic enzymes such as Vat promotes mucosal invasion [21]. The adhesion to and invasion of IECs is mediated by the bind of FimH adhesin of type I pili to the Carcinoembryonic Antigen-related Cell Adhesion Molecules 6 (CEACAM6) receptor, which indeed is upregulated in CD patients [29]. On the other hand, some AIEC strains have FimH adhesin variants that more efficiently bind IECs [30]. OmpC [31], ChiA [32], and the flagella [33] are also important for AIEC to adhere to IECs.

An outstanding feature of AIEC is their ability to survive inside macrophages [20], and genes such as *hrtA* [34], *dsbA* [35], *ibeA* [36], and *hfq* [37] are important for AIEC intramacrophage fitness, along with the SOS and stringent responses [38], the capacity to form biofilm-like structures within phagolysosomes to avoid lysis [39], and the ability to switch to a non-replicative state after phagocytosis, with a fraction of the population becoming persister cells [38].

## 3. Putative TA Roles in AIEC’s Pathogenesis

The array of TAs in AIEC became known some time ago [9] with the revelation that the NRG857c chromosome contains a minimum of 33 TAs belonging to types I, II, IV, and V. Some of these TA toxin genes exhibited in vitro responsiveness to bile salts and acid stress, as well as to conditions found within macrophages [9]. A recently updated online TA database, TADB 3.0 [40], reports 16 and 20 TAs in LF82 and NRG857c genomes, respectively, in both the chromosome and extrachromosomal plasmids (Table 1 and Table 2). Discrepancies in NRG857c between both sources are due to non-annotated genes that are not contained in TADB 3.0, as well as novel genes (type VIII SdsR-RyeA) or plasmid systems not considered in the first report [9]. Nevertheless, these findings suggest that AIEC possess a responsive arsenal of TAs, which are intriguing bacterial factors whose exploration can deepen our comprehension of AIEC’s pathogenesis and their involvement in the chronicity of CD.

TAs reportedly control a broad range of cell transition states in response to various environmental stresses and are involved in different biological processes. However, there are four currently accepted bona fide TA roles in bacterial cell physiology, including growth diminution during stress, phage inhibition, MGE maintenance, and biofilm formation [2]. All of them may play a role in AIEC’s pathogenesis and are discussed here.

### 3.1. Roles in Stress Response

Although the physiological role of TAs as stress response modules is questioned [5], there are several reports of TAs playing important roles in pathogenic bacteria activity in response to various environmental stress, with not all TAs responding to the same stress [8,10,41].

The presence of bile salts is a host signal encountered by enteric bacteria as they travel through the gastrointestinal tract, and enteric pathogens utilize bile as a signal to modulate virulence factor expression [42]. Accordingly, in the presence of bile salts, AIEC induce long polar fimbriae expression to allow the bacteria to interact with Peyer’s patches and M cells [43]; they undergo metabolic adaptations [23], and some of their chromosomal TA toxins are upregulated [9]. Homologs of toxin genes *yafO*, *parE*, *hipA*, *mazF*, *yoeB*, *cptA*, and *ortT*, are upregulated in response to bile salt treatment in both NRG857c and another phylogenetically distant AIEC strain, HM605 [9,44].

In addition to bile salts, AIEC encounter diverse stressful conditions during host infection, as the intracellular environment of macrophages is a great threat to survival. In this setting, AIEC must face acid stress and oxidative stress, toxic metal cations, and antimicrobial peptides. Under in vitro acidic conditions and intramacrophage conditions, AIEC respond, inducing expression of an arsenal of toxin genes, such as *ccdB*, *yafO*, *parE*, *yoeB*, *mazF*, *cptA*, *ghoT*, and *ortT* [9]. As TA genes can respond to different stress conditions, activation of different toxin genes could be triggered by diverse intramacrophage stresses, collectively contributing to AIEC survival. Indeed, toxin genes upregulated within the macrophage, such as *mazF*, *cptA*, and *ortT*, do not necessarily respond to acid stress in vitro [9], which suggests that they could be responsive to different stress in the intramacrophage environment.

CcdB toxin targets DNA gyrase, affecting bacterial DNA replication [45]; it belongs to the CcdAB system, which is well known for ensuring F-plasmid maintenance [46]. Its chromosomal counterpart in pathogenic *E. coli* O157:H7 has been found to contribute to antibiotic tolerance [47]. C-terminal residues W99, G100, and I101 are important for CcdB toxicity but not for its regulatory function as a transcriptional regulator of its own operon (as part of the TA complex with its cognate antitoxin CcdA) [48]. AIEC NRG857c harbors a CcdB W99D variant, identical to its homolog from the ExPEC CFT073 strain [9], but it is unknown if these are bona fide TA toxins. However, in *Salmonella*, a CcdB W99R variant is known to be non-toxic but is still expressed in vitro and within the host [41]. Similarly, in AIEC NRG857c, the *ccdB* gene is upregulated in bile salt and acidic stress conditions, as well as within macrophages [9]. As suggested for *Salmonella* [41], these CcdB toxins may be diverging and losing properties compared to their functional homologs, highlighting that their contributions to bacterial pathogenicity should be tested.

YafNO, ParDE, and YefM/YoeB all belong to the TA *relBE* family, a diverse family whose members are classified by similarities in secondary and/or tertiary structures [49], although they may have different properties and respond differentially to environmental stresses [49,50]. In AIEC, the exposure to bile salt and acidic stress conditions upregulates the *yafO, parE*, and *yoeB* homologs [9].

YafO is a ribosome-dependent mRNA interferase that inhibits protein synthesis [51]. *E. coli yafO* toxin gene is induced by several stressful conditions, including antibiotic treatment, amino acid starvation, and glucose starvation [50]. *yafO* is also upregulated by the SOS response [52], which is crucial for AIEC survival inside the macrophage [38]. Consequently, *yafO* is highly upregulated in response to bile salts and stresses inside the macrophage [9].

ParE is a DNA gyrase inhibitor that blocks DNA replication [53]. Ectopic expression of *parDE* from conjugative IncI and IncF plasmids found in *E. coli* and *Salmonella* species was observed to promote biofilm formation in *E. coli* [54]. NRG857c *parE* genes are encoded downstream of *higA* genes, which appear to be their cognate antitoxins. The HigBA system, also a member of the *relBE* family, is involved in tolerance to bile salts in the Gram-positive bacteria *Weissella cibaria*. When bacteria were exposed to bile salts, HigBA was activated, and persister cells were formed to escape the stress, improving viability [55]. In addition, HigBA was shown to be a growth regulator during DNA damage stress in *Caulobacter crescentus* [56]. Although they remain to be characterized, *parE*/*higA* TA pairs could be bona fide TAs and play a role as stress response elements in AIEC.

YoeB is a ribosome-dependent mRNA interferase involved in the stress response of different pathogens. In *E. coli*, YoeB is activated during thermal stress without eliciting growth arrest [57]. In the Gram-positive *Streptococcus pneumoniae* and the aquatic *Edwardsiella piscicida* pathogens, a *yefM*-*yoeB* deletion reduces the response to oxidative stress [58,59], while in the ExPEC isolate CFT073, *yefM*-*yoeB* enhances bladder colonization [8]. During their intramacrophage lifestyle, AIEC must face acidic and oxidative stresses to survive; in consequence, *yoeB* expression is induced under acidic stress and is one of the most expressed toxin genes inside macrophages [9].

Altogether, antecedents of the NRG857c and HM605 strains and other pathogens suggest that *relBE* members probably affect AIEC’s response to the diverse stressful conditions found during host infection.

Another *relBE* member corresponds to the MqsRA system, which influences *E. coli* during bile acid stress [60] and whose antitoxin MqsA is considered a regulator of other cell regulators [61]. However, the role of MqsRA in stress response has been questioned [62]. Wang et al. [61] proposed that MqsA regulates the general stress response through the direct transcriptional repression of the stationary phase sigma factor RpoS, reducing metabolism through mRNA decay and activating type V toxin GhoT. Homologous MqsRA was not identified in NRG857c or LF82; indeed, *ghoT*, a target regulated by MqsA, was downregulated in response to bile salts [9]. Conversely, the GhoT-related orphan toxin gene, *ortT-1*, was highly upregulated in these conditions and in different AIEC strains [9]. In *E. coli*, OrtT was found to be important for maintaining cell fitness during stringent stress, diminishing both growth and metabolism [63]. As AIEC rely on their stringent response to survive inside the macrophage, OrtT toxin, as well as other TA toxins, may be important to triggering survival strategies in this intracellular environment, as discussed below (see Section 3.5.2).

In *E. coli*, the type IV CptAB system comprises a membrane-associating toxin, CptA, that inhibits cell division by interfering with the polymerization of cytoskeletal proteins [64]. However, homologs in *Serratia* sp. strain ATCC 39006 [65] and *Shewanella oneidensis* [66] are not part of a bona fide TA system, although the genes conserve synteny and a CptA homolog still might interact with multiple cell division proteins. The homologous antitoxin CptB in *S. oneidensis* is required for normal growth and contributes to stress tolerance [66]. In AIEC, the *cptAB* system identified in silico (Table 1 and Table 2, [9]) is identical to the one characterized by Masuda et al. [64], and a homologous *cptA* toxin gene is slightly upregulated under bile salt and acidic stress, as well as inside macrophages [9]. On the contrary, in *Acinetobacter baumannii*, *cptAB* genes are downregulated under oxidative and antibiotic stress [67]. This emphasizes the variability within TA systems and that each system must be studied considering its natural genetic context to decipher its contribution to bacterial physiology.

Different TAs may independently provide significant advantages to AIEC within specific host environments where they must deal with diverse stresses. However, although the role of TAs in stress response has been questioned [5,62], it is important to characterize them in their native strain’s background and biological context, especially in pathogens like AIEC. Here, it is relevant to consider AIEC’s special features, which contrast with those of other *E. coli* strains, to uncover the real contribution of TAs to stress response, physiology, and pathogenicity.

### 3.2. Roles in Biofilm Formation

Biofilms consist of organized bacterial communities embedded within polysaccharide polymers, providing protection against antibiotics and evasion of host innate immunity; they can exist both extracellularly and intracellularly. Interestingly, biofilm formation can be a strategy to sustain intracellular bacterial populations inside host cells, contributing to persistence [39]. Moreover, microbial biofilms are often linked to chronic diseases like IBD [68]. Several studies indicate that TAs may influence biofilm development, although the exact mechanisms remain unclear.

AIEC are known biofilm producers [39,69] and possess TAs that can be involved in biofilm formation. In particular, AIEC possess homologs of TA *mazEF*, *hipBA*, *ccdAB*, *higBA*, *yefM-yoeB*, and other *parE* toxins (Table 1 and Table 2) that are reportedly involved in biofilm formation in other bacteria.

MazF, the toxin component of TA MazEF, is a ribosome-independent sequence-specific endoribonuclease [70]. The deletion of *mazEF* was observed to reduce biofilm formation in *E. coli* MC4100*relA+*, and it was suggested that TA-mediated cell death was important for optimal biofilm formation [71]. In contrast to its role in *E. coli*, *mazF* is proposed to inhibit biofilm formation and promote biofilm antibiotic tolerance in *Staphylococcus aureus* [72]. Moreover, the deletion of *mazEF* along with four more TAs (*relBE*, *chpB*, *yefM-yoeB*, *dinJ*-*yafQ*) influenced biofilm formation in a temporal manner in *E. coli* MG1655 (less biofilm formation at 8 h and more biofilm formation at 24 h). Deleting these five TA systems promotes the expression of *yjgK*, an uncharacterized protein that represses fimbria genes in *E. coli* MG1655 [73]. On the other hand, the deletion of each of these five toxins independently (*mazF*, *relE*, *chpB*, *yoeB*, and *yafQ*) increased early biofilm formation while overexpression of the toxins repressed biofilm formation in *E. coli* BW25113, suggesting the role of the antitoxins in the regulation [73].

RelE is a ribosome-dependent codon-specific endoribonuclease [74], and deletion of *relBE* also reduced biofilm formation in *E. coli* K12 [75]. Similar to the role in *E. coli*, deletion mutants of *relBE* systems formed significantly less biofilm than the wild-type strain in *Vibrio cholerae*, and deletion mutants of *relBE* and *yefM*-*yoeB* also decreased the biofilm formation in *Streptococcus pneumoniae* [58,76]. It has been suggested that the RelBE family influences the entire process of biofilm development in *V. cholerae* because different *relBE* deletion mutants decreased biofilm formation at different stages of biofilm development [76]. However, *mazF*, *relE*, or double deletion mutants had no effect on biofilm formation in *Streptococcus mutants* [77]. Moreover, *yefM*-*yoeB* inhibited biofilm formation in *Edwardsiella piscicida* [59], and overexpression of toxins resembling RelE and VapC in *Burkholderia cenocepacia* showed a positive effect on biofilm formation [78]. In addition, ectopic expression of *parDE* TA promoted biofilm formation in *E. coli* [54], and deletion of *parDE* in *Caulobacter crescentus* increased biofilm formation [79].

Homologs of *mazEF*, *relBE*, and *yefM-yoeB* are encoded by AIEC, and we can speculate on their similar roles in biofilm formation. In AIEC, these systems are induced in the presence of bile salts [9], a condition that also increases the transcription of genes involved in biofilm formation [23]. Notably, enteric pathogens like *Salmonella* and *Shigella* form a biofilm in the presence of bile salts [42,80], which might favor gut colonization.

*higAB* was found to play no role in biofilm formation in *E. coli*. In *Pseudomonas aeruginosa*, HigB toxin from the *higAB* system reduces biofilm formation by reducing the intracellular levels of c-di-GMP, which, in turn, induces motility [81,82]. In addition, the deletion of *higAB* and *higB* influences biofilm formation in *Edwardsiella piscicida* [83].

HipA is a serine/threonine kinase that phosphorylates elongation factor thermal unstable (EFTu) and inhibits protein synthesis [84]. *hipBA* is reported to be involved in the production of eDNA, an important structural component of biofilms in *E. coli* [85]. In line with this, transcriptional silencing of *hipBA* and *ccdAB* significantly reduced biofilm formation in the probiotic strain *E. coli* Nissle 1917 [86]. Homologs of *hipA* and *ccdB* are induced in AIEC both in the presence of bile salts and inside macrophages [9], conditions where biofilm formation is important to successfully colonize and persist.

As noted, several TAs could influence the development of biofilm formation in AIEC. Given the significance of biofilm formation in AIEC pathogenicity, exploring the involvement of TAs in this process represents a novel and clinically relevant area of research.

### 3.3. Role as Phage Inhibition Systems

Bacteriophages reside within the gut environment and form a major part of the gut microbiota. They selectively infect bacterial strains and naturally aid the maintenance of the gut microbiota and its composition [87]. There is growing evidence that TAs play critical roles in protecting bacteria against bacteriophages, and these systems are thought to mediate abortive infection, wherein the host cell dies in response to phage infection [88].

AIEC are susceptible to phage infection and possess TAs as part of their genomic repertoire, which may be involved in defending against phage predation [9]. Type II *mazEF* system and type I *hok*/*sok* system, which are included in the AIEC TA repertoire, were reported to participate in phage defense in *E. coli*. *mazEF* was described to mediate cell death as a phage P1 defense mechanism [89]. However, the role of *mazEF* remains uncertain because these results could not be replicated [90]. Moreover, the *hok*/*sok* system from the R1 plasmid was found to protect against the T4 phage in *E. coli* K12, although the mechanism is still not clear [91].

Prophages are not unusual on AIEC genomes. While some defective prophages are present on NRG857c [18], LF82 encodes five prophages considered to be complete and functional [25], and homologs have been identified in contigs from *E. coli* isolated from CD patients [92]. However, phage resistance assays performed with *E. coli* isolated from CD patients revealed that sensitivity or resistance to some tested phages was not necessarily related to the presence or absence of a particular prophage in a genome [92], which suggests a putative role for TAs in mediating abortive infection in AIEC. On the other hand, it has been hypothesized that the survival of LF82 in macrophages is partly due to its ability to control the induction level of its most active prophage [93]. Notably, inside macrophages, AIEC express an arsenal of TA toxin genes [9], and we can speculate on the role of TAs in controlling prophage induction levels in these conditions by a mechanism similar to abortive infection. Overall, TA’s activation could contribute to the survival and persistence of AIEC within the gut environment and macrophage by providing phage defense mechanisms.

In recent years, phage therapy has regained attention as a therapeutic approach to combat infectious diseases [94]. Bacteriophages that target AIEC were found to reduce DSS-induced colitis symptoms in CEABAC10 transgenic LF82-colonized mice and significantly reduce the number of AIEC in feces and in the adherent microbiota of intestinal sections [95]. Therefore, phages targeting AIEC strains are a promising new treatment for IBD, and elucidating whether their TAs mediate abortive infections is crucial for the design of efficient bacteriophage therapies.

### 3.4. Roles in MGEs Maintenance

Although TAs are nearly ubiquitous within bacterial genomes, individual TAs exhibit restricted gene synteny, and they are commonly part of the accessory genome. Indeed, TAs were originally discovered on plasmids and associated with the PSK of plasmid-free cells [4], leading to plasmid maintenance in the bacterial population. Nowadays, it is recognized that, regardless of their location in plasmids or chromosomes, TAs may influence the maintenance of genetic elements to which they are physically linked, such as genomic islands (GIs) [96,97,98,99]. This maintenance role may influence the host-adapted lifestyle and evolution of important pathogens [100].

Plasmids play a critical role in enabling bacteria to adapt to specific environments and stresses, as they often carry genes that confer resistance to antibiotics and/or genes associated with pathogenicity [101,102]. AIEC LF82 and NRG857c harbor different extrachromosomal plasmids, whose contribution to AIEC evolution and pathogenicity remains to be investigated. Nevertheless, plasmids seem to be ubiquitous in AIEC, as sequencing data from AIEC clinical isolates retrieved plasmid contigs similar to sequences of plasmids from pathogenic bacteria, e.g., UPEC and *Salmonella* [92].

AIEC LF82 contains a plasmid of size 108,379 bp (plLF82), which may be acquired via horizontal gene transfer from *Yersinia* or *Salmonella* [25]. plLF82-homologous sequences in CD-associated *E. coli* have also been identified [92]. A different plasmid is carried by AIEC NRG857c (pO83_CORR, 147,060 bp), resembling an antimicrobial multi-resistance plasmid [18]. According to the database TADB 3.0 [40], plLF82 is devoid of TAs, while pO83_CORR possibly carries at least three putative TAs belonging to type I *hok-sok* and type II VagCD and VapBC families (Table 2).

The *hok/sok* locus is well known for its plasmid stabilization function, but it also affects growth control and may complement the existing or defective SOS mechanism [103]. Besides being chromosomally encoded, VapBC and VagCD TA systems are abundant on plasmids from different bacterial pathogens [104,105], where they might participate as plasmid maintenance modules. VapC and VagD toxins belong to the PIN-domain family of proteins and inhibit translation through the cleavage of RNAs [106]. On *Shigella*, a large virulence plasmid, pINV, critical for virulence, relies on a member of the VapBC family (MvpAT) to ensure its retention inside the host [107]. In consequence, type I *hok/sok* and VapBC (VagCD) in pO83_CORR may function similarly to their plasmidial homologs in other bacteria, which would enhance the ability of AIEC NRG857c to establish infections and propagate the antibiotic resistance elements carried on pO83_CORR, thereby contributing to its pathogenicity.

Besides plasmids, GIs are mobile elements known to contribute to bacterial fitness and could encode genes involved in pathogenicity. AIEC carry different GIs not exclusive to the pathotype. For instance, 9 large GIs were identified in the LF82 genome [25] and 35 GIs on NRG857c, which are also highly conserved in LF82 [18], suggesting that they may encode traits relevant to the AIEC pathotype.

HipBA and PemKI type II TAs were identified on chromosomal MGEs in AIEC (Table 1 and Table 2). HipBA (*hipBA-1*, TA21 at [9]) is encoded close to a variable region upstream of an F9 fimbrial operon. This *hipBA* locus is conserved between non-pathogenic and pathogenic bacteria; in addition, a complete F9 operon is only present in pathogenic *E. coli* [9,108]. In UPEC, F9 fimbrial expression is regulated by H-NS and temperature; it plays a role in biofilm formation [108,109], and it provides a fitness advantage during inflammatory conditions in a mouse model [110]. The regulation and contribution of the F9 operon to AIEC pathogenicity are unknown, along with the contribution of the *hipBA* locus. For instance, in AIEC NRG857c, *hipA-1* is upregulated under in vitro stress conditions and those found inside macrophages; on the other hand, it is completely switched off inside macrophages in the HM605 strain [9]. Differential expression regulation between AIEC strains and TAs highlights the diversity of both the AIEC pathotype and TAs, and it underscores the importance of studying the biological roles of TAs in their specific genetic contexts.

PemKI was originally considered responsible for the stable maintenance of plasmid R100 [111] but is now known to have different roles in several plasmids and bacteria [112,113]. In AIEC NRG857c, the *pemKI* locus (*mazEF-1*, TA22 at [9]) is encoded within an MGE containing the genes of a phosphoenolpyruvate-dependent sugar phosphotransferase system [9]. PemKI is known to be plasmid-encoded, its chromosomal counterpart being the MazEF system (also known as ChpBA) [114]. However, several *pemKI* loci have been found on plasmids and chromosomes from different bacteria [115]. These recall the mobile nature of TAs, which can jump from extrachromosomal elements to the chromosome or within the same chromosome. However, the functionality of these systems must be tested. Of note, Janczak et al. [116] reported that the location of a *pemKI* locus in the bacterial chromosome results in the loss of its toxicity. Thus, it is important to test whether AIEC’s mobile TAs are bona fide systems and how they contribute to bacterial pathogenicity.

### 3.5. Role in the Host Lifestyle

AIEC’s main pathophysiological features are the adhesion to and invasion of IECs and replication inside macrophages without inducing cell death [15,19,20]. Although some genetic factors were shown to be important for these intracellular lifestyles, much remains to be revealed. TAs were reported to be involved in bacterial pathogenicity, including intracellular survival [41,117]. Indeed, different TAs control the *Salmonella* lifestyle inside eukaryotic cells [41]. For AIEC, distinct microenvironments found inside different eukaryotic cells could trigger disparate arsenals of TAs. In consequence, AIEC could rely on their TA repertoire to ensure their intracellular survival, which is critical for the progression of the infection, as is discussed here.

#### 3.5.1. Intra-IECs Lifestyle

To date, both type I and II TAs have been reported to influence bacterial intracellular survival within epithelial cells, though only a few studies have been performed on epithelial cells of intestinal origin. Within IECs, LF82 is in vacuoles or free in the host cell cytoplasm [19]; however, besides some transcriptomic studies of AIEC during in vitro infection [118], its physiological status inside IECs remains unknown. Further research on the role TAs might play in IECs’ intracellular survival is necessary.

The AIEC array of TAs previously reported to be involved in intracellular eukaryotic lifestyles in other bacteria includes *hok*, *ldrA*, and *higB* toxins (Table 1 and Table 2).

Several studies performed on the well-known intracellular pathogen *Salmonella* report that TAs control bacteria lifestyle inside eukaryotic cells. A proteomic and expression analysis confirmed that intracellular *S.* Typhimurium produces functional toxins encoded by type I (Hok, LdrA, and TisB) and type II (T2_ST_, T4_ST_, T5_ST_ and VapC2) TAs in fibroblasts. Deletion mutants of *hok*-*sok*_ST_, *ldrA*-*rdlA*_ST_, *tisB*-*istR*_ST_, *ta4*_ST_, and *vapBC2*_ST_ showed reduced intracellular survival inside fibroblasts [41]. Notably, only the *vapBC2*_ST_ deletion mutant showed reduced intracellular survival inside HeLa epithelial cells [41]. Furthermore, the deletion mutant of *hha* and *tomB4* from type II TAs showed reduced invasion ability across HCT116 colon carcinoma cells compared to the wild-type strain in *Salmonella enterica* [119]. The downregulation of the master regulator *hilA* and the reduced expression of *Salmonella* Pathogenicity Island-1 (SPI-1) genes in the mutant strain is proposed to cause the decreased invasion ability [119]. Similarly, Song et al. reported the activation of type II TA PA1030/PA1029 (not yet characterized), PA1878/PA1879 (VapBC homolog), and PA4674.1/PA4674 (denoted as HigBA) during the *P. aeruginosa* infection of A549 lung epithelial cells. Deletion mutants of these TAs showed no difference in the adhesion ability to A549 epithelial cells compared to the wild-type strain, whereas the *higB* mutant strain showed reduced invasion ability [120]. Other studies performed using a model of the primary human upper airway tissue indicated that deletion mutants of type II TA *toxAvapA*, *vapBC-1*, *vapXD* had lower intracellular survival levels over the 8 days of co-culture compared to the wild-type strain in non-typeable *Haemophilus influenzae* [121,122].

On the other hand, TA disruption was also found to increase intracellular survival. Disruption of the *fit* (fast intracellular trafficker) locus (*fitAfitB*) TA caused an accelerated replication rate of *Neisseria gonorrhoeae* within the A431 epithelial carcinoma cell line and T84 colonic carcinoma cell line and a quick transit through the polarized T84 epithelial monolayer compared to the wild-type strain [123].

The role of TAs in intracellular survival has been demonstrated in several bacterial species, though it is very limited in *E. coli*. Deciphering the role TAs may play in intracellular survival and replication within IEC could lead to the design of new therapies for CD patients colonized by AIEC.

#### 3.5.2. Intra-Macrophage Lifestyle

The hallmark of AIEC is their capacity to survive longer and replicate within macrophages [20]. In this intracellular environment, LF82 remains in mature phagolysosomes [35] and forms biofilm-like structures [39] to confront several stresses. As we have noted, the stress response is key to the survival of AIEC in macrophages, and some genes have been identified supporting AIEC survival and replication in this niche, including *htrA*, *dsbA*, *ibeA*, and *hfq* [34,35,36,37], as well as the SOS and stringent responses [38].

The role of TAs in the survival of bacteria inside macrophages has been studied almost exclusively in *Salmonella* Typhimurium. *S.* Typhimurium strain 12023s cells enter a non-growing state upon entry to host macrophages through a mechanism dependent on type II TAs [124]. Fourteen type II TA operons were upregulated within 30 min of *Salmonella* phagocytosis, and although their deletion did not impair intracellular replication rates, the deletion of these TA genes reduced the proportion of nonreplicating bacteria in infected macrophages [124]. TacT, a novel GCN5-related N-acetyltransferase (GNAT)-like toxin, was responsible for promoting the *Salmonella* non-growing state through acetylation of tRNA [125]. The participation of different GNAT toxins in the macrophage survival of clinical invasive strains of *S.* Typhimurium and Enteritidis was later revealed [126]. In addition to GNAT toxins, Rhs toxins were shown to comprise functional type II TAs and to affect the proliferation of *Salmonella* during macrophage infection [127].

On the other hand, in *Enterococcus faecalis*, the type I ef0409-ef0408 system was shown to be involved in the infection process and survival inside host cells. A *Δef0408* antitoxin mutant exhibited a hypervirulence phenotype in the infection model *Galleria mellonella* and in macrophages, as the mutant survived better than the wild-type [128]. Michaux et al. [128] hypothesized that free toxin ef0409 might have contributed to the hypervirulent phenotype and that ef0408 sRNA could be acting as a sensor and suppressor of ef0409 toxin activity to control growth and virulence. They proposed an equilibrium between favorable colonization (by repressing virulence) and pathogenicity according to the host environment, and sRNA such as that from type I TA could act as a key regulator in the transition from a commensal relationship to virulence [128,129].

As in *Salmonella* [124], an arsenal of TAs is upregulated in AIEC after phagocytosis [9]. However, GCN5 toxins have not been identified in AIEC, and currently, no Rhs toxin is characterized in this pathotype, meaning different toxins should be involved in the growth regulation of AIEC within macrophages. Among them, *ccdB*, *yafO*, *hipA*, *parE*, *yoeB*, *mazF*, *cptA*, *ghoT*, and *ortT* are putative candidates as they are highly upregulated after AIEC phagocytosis [9].

TAs may participate in different processes important for AIEC intramacrophage survival. For instance, they could serve as stress response modules (See Section 3.1), in the formation of intracellular biofilm-like structures (see Section 3.2), and as phage inhibition systems (see Section 3.3). However, forming persister cells is also a strategy of AIEC to avoid macrophage killing and favor survival [38], and the participation of TAs in this process is discussed in the following subsection.

### 3.6. Roles in Persister Cell Formation

Bacterial persistence corresponds to a reversible phenotypic state in which a small subpopulation of bacteria remains non-replicative, which allows them to survive deadly stress conditions such as antibiotic treatments. In consequence, persister cells hinder the treatment of bacterial infections and chronic diseases [130] like CD. For instance, AIEC rely on their biofilm and persister cell formation to acquire the maximum protection against macrophage attack or antibiotics [131], which could allow them to establish a long-term survival niche within phagocytic cells in CD patients, making treatment more challenging.

Persister cells are produced spontaneously or stress-induced; however, the molecular mechanisms underlying their formation remain elusive. Activations of stringent response through (p)ppGpp [132,133], SOS response [134], and TAs [124,125] are some of the molecular mechanisms linked to persister cell formation. Nevertheless, the participation of TAs is controversial [5,135,136,137], in part because some experiments were conducted in experimental conditions not relevant to TA activation. Of note, certain TA mutants displayed altered levels of persisters in a specific biological context but not when they were studied in in vitro laboratory conditions.

Remarkably, the first gene associated with persistence corresponded to a TA toxin, *hipA* [138]. In *E. coli* HipA corresponds to a serine/threonine-protein kinase that phosphorylates glutamyl-tRNA-synthetase, leading to the accumulation of uncharged tRNA-Glu in the cell and the consequent activation of stringent response [139]. A variant HipA7 (G22S and D291A) is non-toxic and associated with a high-persistence phenotype in *E. coli* [132]. Similarly, a homologous HipA was associated with persistence in *Caulobacter crescentus* [140].

TAs other than *hipA* have been shown to contribute to persistence in several bacterial species, for example, *yafQ/dinJT*, *tisAB/istR*, *hokB/sokB*, and *mqsRA* in *E. coli* [141,142,143,144]; *tacT* toxin, *relBE*, *parDE*, *higBA*, *and vapBC* in *Salmonella* [124,125]; and *smuATR* in *S. mutans* [145]. Recently, Ma et al. described the role of the MazEF system in *S. aureus* chronic infection [72]. They examined MazEF in virulence using a murine model and found that *mazF* increases antibiotic tolerance and allows bacteria to transition from acute to chronic infection [72].

AIEC encode homologs of TAs previously reported to be involved in persister cell formation. While homologs of *tacT*, *yafQ*/*dinJT*, and *mqsRA* were not identified, *hok/sok*, *relBE*, *higBA*, *vapBC*, *mazEF*, and *hipA* genes are present on AIEC genomes (Table 1 and Table 2). Of them, *mazF* and *hipA* were shown to be upregulated inside macrophages [9], where AIEC persister cells increase [38]. Curiously, *hipA* homologs were highly upregulated in NRG857c under conditions found inside the macrophage but completely repressed in HM605 [9]. An AIEC HipA homolog lacks the described mutations of the high-persister HipA7 variant but instead shares some amino acid variants with its phylogenetically close ExPEC CFT073 [9].

Undoubtedly, further research on the effect TAs might have on AIEC persistence will be meaningful, with special attention paid to the contribution of *hipA* and its variants.

## 4. Conclusions

Bacteria are constantly evolving to improve their fitness and pathogenicity, so discovering new targets for antimicrobial strategies is vital in the fight against bacterial pathogens involved in infectious and chronic diseases. Conversely, understanding the mechanisms and virulence factors contributing to different pathogenesis stages may enable the rational and successful development of new treatments for those diseases.

For AIEC, potential therapeutic strategies include targeting bacterial colonization of gut mucosa using phage therapy, bacteriocins, and anti-adhesion molecules, as well as genetically engineered microbes as biosensors or delivery vehicles to potentially deliver therapeutics to disease sites [146]. However, given the special lifestyle of AIEC, which differs from that of other *E. coli* pathotypes, strategies to target intracellular bacteria should also be considered.

**Figure 1:**
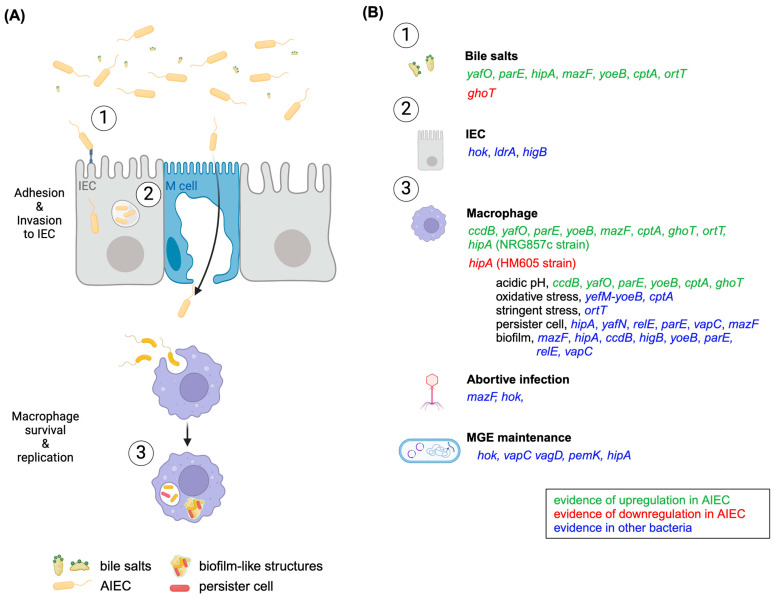
Overview of AIEC’s pathogenic route and the TAs that could contribute to pathogenicity: (**A**) AIEC must outcompete the native microbiota and challenge exposure to bile salts to successfully colonize the intestinal mucosa. There, AIEC can adhere to and invade IEC, where they stay in vacuoles, as well as translocate across M cells and colonize macrophages. Once phagocytosed, AIEC form biofilm-like structures and persister cells within phagolysosomes to survive. (**B**) TAs could affect different stages of the AIEC pathogenicity route shown in (**A**) and successfully confront stress conditions. For instance, TAs could be involved in the AIEC bile salt response (number 1), IEC (number 2), and macrophage (number 3) lifestyles. Inside macrophages, TAs could be involved in the AIEC response to acidic pH, oxidative and stringent stress, and the formation of persister cells and biofilm-like structures. In addition, the roles TAs might play in abortive phage infection and MGE maintenance could also be important for AIEC pathogenicity, although there is no clear evidence regarding which pathogenicity stage they might affect. The circled numbers indicate where TAs could be involved in the scheme in (**A**). TA toxin gene names are given for those with evidence in the literature [9] and colored according to the legend. Figure created with BioRender.com.

TAs have arisen as novel therapeutic targets [3] partly because there are no eukaryotic homologs, and TAs produce toxins that are not secreted but instead act only within the producing cell, disabling their microbial host from the inside. As we have reviewed here, TAs can have a role in different stages of AIEC pathogenicity, for instance, stress response, biofilm formation, phage inhibition, MGE maintenance, and persister cell formation; all are also important for AIEC intracellular lifestyles (Figure 1). Therefore, understanding the contribution of TAs to AIEC physiology and pathogenicity is meaningful.

In a previous study, the expression of an AIEC toxin array was determined in different stress conditions, such as bile salts, acidic pH, and inside macrophages [9]. However, we lack functional studies assessing the role of the toxins since the transcription of a TA does not indicate activity [147]. Moreover, since genetic context and growth conditions will undoubtedly affect the possible biological role of a TA, the contribution of TAs to AIEC pathogenicity should be verified in light of their special growth characteristics, which are different from those of laboratory *E. coli* strains and other *E. coli* pathotypes, in which most TAs were previously characterized.

Undoubtedly, although more research is needed, TAs have the potential to contribute to AIEC pathogenicity, with roles in different stages. As AIEC face changing stress conditions inside the host, each encoded TA could be activated and contribute to their fitness in different ways.

## Figures and Tables

**Table 1:** TA loci of the AIEC LF82 reference strain according to TADB 3.0 [40].

TA ID	Toxin	Antitoxin	Family/Domain	Comments
TA214828	K8B90_RS03460 (symE)	-(symR)	symER/SymE (toxin)	Type I
TA214832	K8B90_RS03850 (hokC)	-(sokC)	hok-sok/-	Type I
TA214852	K8B90_RS22020 (ldrD)	-(rdlD)	ldrD-rdlD/Ldr (toxin)	Type I
TA214826	K8B90_RS00850 (higB)	K8B90_RS00855 (higA)	higBA (relBE)/HTH (antitoxin)	Type II
TA214834	K8B90_RS04020 (ccdB)	K8B90_RS04015 (ccdA)	ccdAB/CcdA (antitoxin)	Type II
TA214835	K8B90_RS05120 (yafO)	K8B90_RS05115 (yafN)	yafN-yafO (relBE)/YafO-YafN	Type II
TA214836	K8B90_RS06040 (Hha)	K8B90_RS06045 (TomB)	Hha-TomB/-	Type II
TA214837	K8B90_RS11255 (higB)	K8B90_RS11250 (higA)	higBA (relBE)/HigB-HigA	Type II
TA214838	K8B90_RS11415 (hipA)	K8B90_RS11420 (hipB)	hipBA/HipA-HipB	Type II; related to MGE
TA214847	K8B90_RS14005 (pemK)	K8B90_RS14010 (pemI)	pemIK/PRK09812-MazE	Type II; related to MGE
TA214848	K8B90_RS14290 (yoeB)	K8B90_RS14295 (yefM)	yefM-yoeB (relBE)/YoeB-YefM	Type II
TA214849	K8B90_RS17940 (mazF)	K8B90_RS17945 (mazE)	mazEF/PRK09907-MazE	Type II
TA214851	K8B90_RS19925 (yhaV)	K8B90_RS19920 (prlF)	prlF-yhaV (relBE)/YhaV-PrlF	Type II
TA214850	K8B90_RS18545 (cptA)	K8B90_RS18550 (cptB)	cptAB/CptA (toxin)	Type IV
TA214827	K8B90_RS02180 (ghoT)	K8B90_RS02175 (ghoS)	ghoTS/ghoT-GhoS	Type V
TA214839	-(SdsR)	-(RyeA)	SdsR-RyeA/-	Type VIII

**Table 2:** TA loci of the AIEC NRG857c reference strain according to TADB 3.0 [40].

TA ID	Toxin	Antitoxin	Family/Domain	Comments
TA027329	NRG857_RS00075 (hokC)	-(sokC)	hok-sok/-	Type I; TA1 at [9]
TA027349	NRG857_RS17965 (ldrD)	-(rdlD)	ldrD-rdlD/Ldr (toxin)	Type I; TA14 at [9]
TA027353	NRG857_RS22450 (symE)	-(symR)	symER/SymE (toxin)	Type I; TA16 at [9]
TA027597	NRG857_RS23200 (srnB)	-(sok)	hok-sok/-	Type I; on plasmid pO83_CORR
TA027602	NRG857_RS23320 (hok)	-(sok)	hok-sok/-	Type I; on plasmid pO83_CORR
TA027331	NRG857_RS00245 (ccdB)	NRG857_RS00240 (ccdA)	ccdAB/CcdA (antitoxin)	Type II; TA17 at [9]
TA027332	NRG857_RS01300 (yafO)	NRG857_RS01295 (yafN)	yafN-yafO (relBE)/YafO-YafN	Type II; TA18 at [9]
TA027333	NRG857_RS02210 (Hha)	NRG857_RS02215 (TomB)	Hha-TomB/-	Type II
TA027334	NRG857_RS07485 (higB)	NRG857_RS07480 (higA)	higBA (relBE)/HigB-HigA	Type II; TA20 at [9]
TA027335	NRG857_RS07640 (hipA)	NRG857_RS07645 (hipB)	hipBA/HipA-HipB	Type II; TA21 at [9]; related to MGE
TA027344	NRG857_RS10225 (pemK)	NRG857_RS10230 (pemI)	pemIK/PRK09812-MazE	Type II; TA22 at [9]; related to MGE
TA027345	NRG857_RS10510 (yoeB)	NRG857_RS10515 (yefM)	yefM-yoeB (relBE)/YoeB-YefM	Type II; TA23 at [9]
TA027346	NRG857_RS13925 (mazF)	NRG857_RS13930 (mazE)	mazEF/PRK09907-MazE	Type II; TA24 at [9]
TA027348	NRG857_RS15890 (yhaV)	NRG857_RS15885 (prlF)	prlF-yhaV (relBE)/YhaV-PrlF	Type II; TA25 at [9]
TA027351	NRG857_RS19860 (higB)	NRG857_RS19865 (higA)	higBA (relBE)/HTH (antitoxin)	Type II; TA27 at [9]
TA027596	NRG857_RS22890 (vagD)	NRG857_RS22885 (vagC)	vagCD/VapC-VagC	Type II; on the plasmid pO83_CORR
TA027601	NRG857_RS23235 (vapC)	NRG857_RS23230 (vapB)	vapBC/VapC-VagC	Type II; on the plasmid pO83_CORR
TA027347	NRG857_RS14535 (cptA)	NRG857_RS14540 (cptB)	cptAB/CptA (toxin)	Type IV; TA31 at [9]
TA027352	NRG857_RS21190 (ghoT)	NRG857_RS21185 (ghoS)	ghoTS/ghoT-GhoS	Type V; TA32 at [9]
TA027336	1894846..1894948 (-) -(SdsR)	-(RyeA)	SdsR-RyeA/-	Type VIII

## Data Availability

No new data were created or analyzed in this study. Data sharing is not applicable to this article.
